# Supplementary material for: Neural Control of Autonomic Arousal During Threat Anticipation Revealed by High-Resolution Cardiac Contractility
Source: bioRxiv. 2026 Feb 24:2026.02.23.707545. Preprint. [Version 1] doi: 10.64898/2026.02.23.707545 (PMC13160061; doi:10.64898/2026.02.23.707545)
Supplement: Supplement 1 [file NIHPP2026.02.23.707545v1-supplement-1.pdf]

bioRxiv preprint doi: https://doi.org/10.64898/2026.02.23.707545; this version posted February 26, 2026. The copyright holder for this preprint (which was not certified by peer review) is the author/funder, who has granted bioRxiv a license to display the preprint in perpetuity. It is made available under aCC-BY 4.0 International license.

Supplementary Information

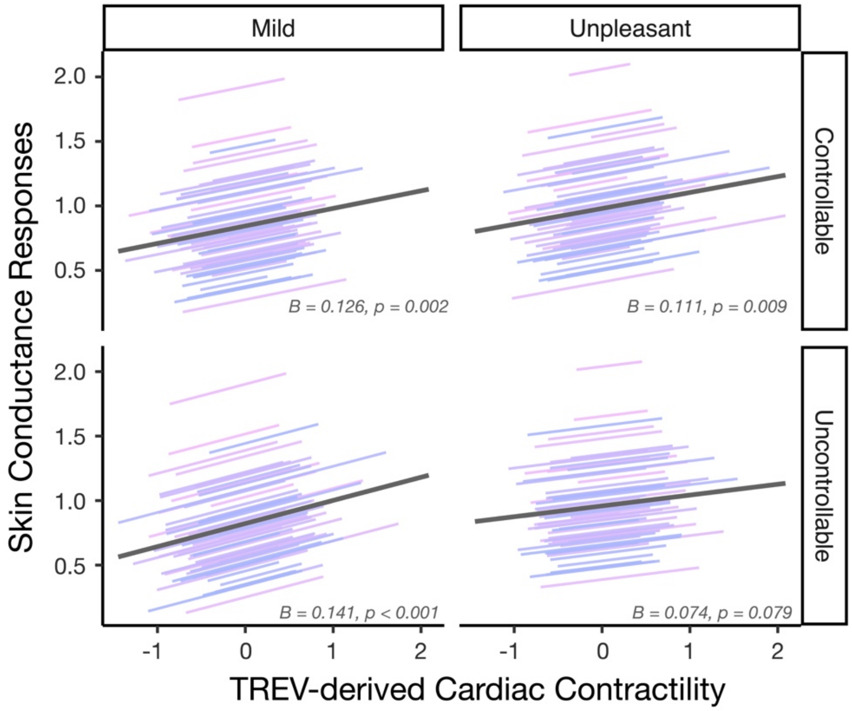

Supplementary Figure 1. TREV and skin conductance responses plotted by task condition. The observed positive association between cardiac contractility and SCRs (reported in the main text) did not significantly interact with threat unpleasantness nor controllability (TREV * threat unpleasantness: p = 0.316; TREV * controllability: p = 0.786; TREV * threat unpleasantness * controllability: p = 0.534).

bioRxiv preprint doi: https://doi.org/10.64898/2026.02.23.707545; this version posted February 26, 2026. The copyright holder for this preprint (which was not certified by peer review) is the author/funder, who has granted bioRxiv a license to display the preprint in perpetuity. It is made available under aCC-BY 4.0 International license.

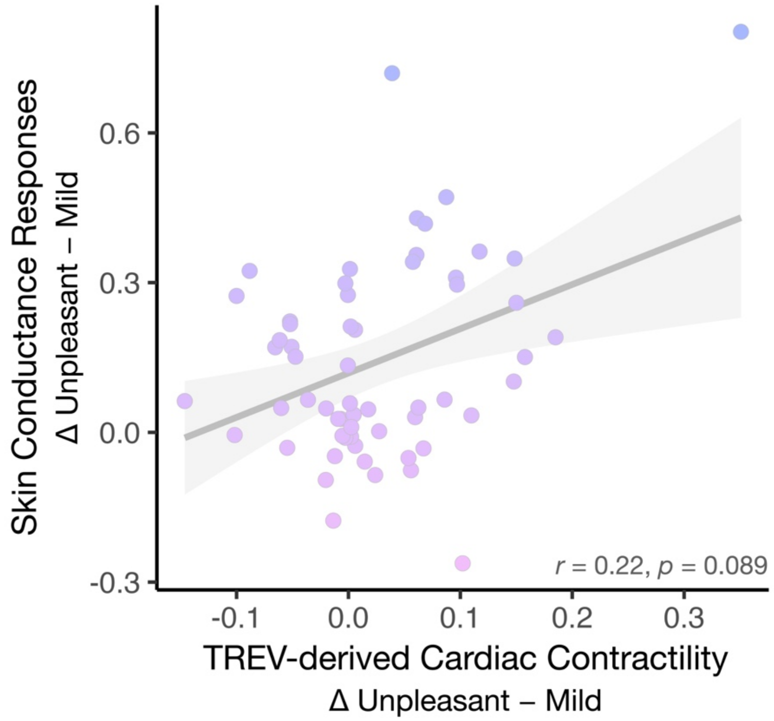

Supplementary Figure 2. TREV and skin conductance responses across participants. Across participants, threat-related changes in TREV-derived cardiac contractility and SCRs were positively but not significantly correlated (*r* = 0.22, *p* = 0.089).

bioRxiv preprint doi: https://doi.org/10.64898/2026.02.23.707545; this version posted February 26, 2026. The copyright holder for this preprint (which was not certified by peer review) is the author/funder, who has granted bioRxiv a license to display the preprint in perpetuity. It is made available under aCC-BY 4.0 International license.

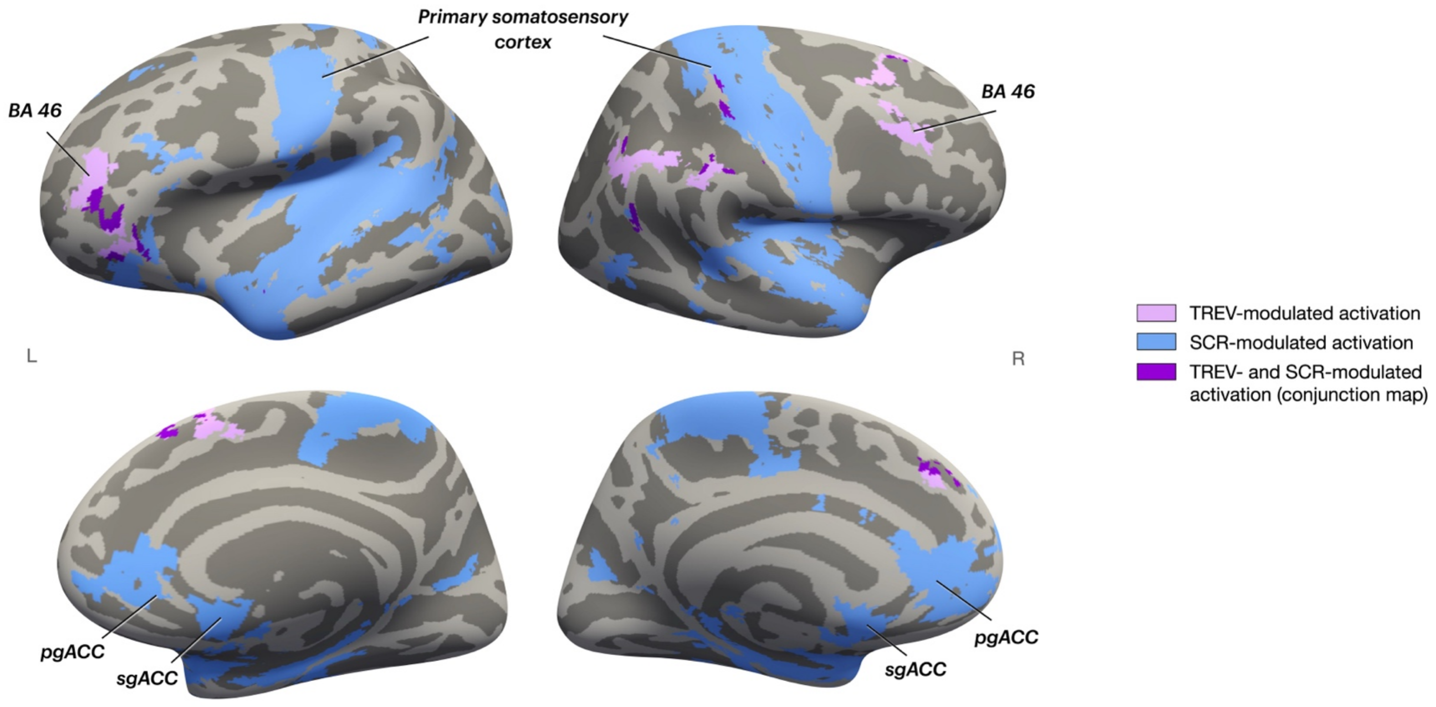

761 **Supplementary Figure 3. Neural correlates of sympathetic responding.** Trial-wise TREV-derived contractility and SCRs were negatively associated with BOLD activation (across task conditions). Significant TREV-modulated betas are shown in lavender, whereas SCR-modulated betas are shown in blue. Clusters of overlapping TREV- and SCR-modulated betas are shown in purple. Whole-brain cluster-level corrected for multiple comparisons at $Z > 2.3$, $p < 0.05$.
